# Native and Invading Yellow Starthistle (Centaurea solstitialis) Microbiomes Differ in Composition and Diversity of Bacteria

**DOI:** 10.1128/mSphere.00088-19

**Published:** 2019-03-06

**Authors:** Patricia Lu-Irving, Julia G. Harenčár, Hailey Sounart, Shana R. Welles, Sarah M. Swope, David A. Baltrus, Katrina M. Dlugosch

**Affiliations:** a Department of Ecology and Evolutionary Biology, University of Arizona, Tucson, Arizona, USA; b Evolutionary Ecology, Royal Botanic Gardens Sydney, Sydney, New South Wales, Australia; c Department of Ecology and Evolutionary Biology, University of California, Santa Cruz, California, USA; d Department of Biology, Mills College, Oakland, California, USA; e School of Plant Sciences, University of Arizona, Tucson, Arizona, USA; f School of Animal and Comparative Biomedical Sciences, University of Arizona, Tucson, Arizona, USA; University of Wisconsin-Madison

**Keywords:** bacterial communities, invasive species, phyllosphere, plant microbiomes, rhizosphere, species introductions

## Abstract

Invasive species could benefit from being introduced to locations with more favorable species interactions, including the loss of enemies, the gain of mutualists, or the simplification of complex interaction networks. Microbiomes are an important source of species interactions with strong fitness effects on multicellular organisms, and these interactions are known to vary across regions. The highly invasive plant yellow starthistle (Centaurea solstitialis) has been shown to experience more favorable microbial interactions in its invasions of the Americas, but the microbiome that must contribute to this variation in interactions is unknown. We sequenced amplicons of 16S rRNA genes to characterize bacterial community compositions in the phyllosphere, ectorhizosphere, and endorhizosphere of yellow starthistle plants from seven invading populations in California, USA, and eight native populations in Europe. We tested for the differentiation of microbiomes by geography, plant compartment, and plant genotype. Bacterial communities differed significantly between native and invading plants within plant compartments, with consistently lower diversity in the microbiome of invading plants. The diversity of bacteria in roots was positively correlated with plant genotype diversity within both ranges, but this relationship did not explain microbiome differences between ranges. Our results reveal that these invading plants are experiencing either a simplified microbial environment or simplified microbial interactions as a result of the dominance of a few taxa within their microbiome. Our findings highlight several alternative hypotheses for the sources of variation that we observe in invader microbiomes and the potential for altered bacterial interactions to facilitate invasion success.

**IMPORTANCE** Previous studies have found that introduced plants commonly experience more favorable microbial interactions in their non-native range, suggesting that changes to the microbiome could be an important contributor to invasion success. Little is known about microbiome variation across native and invading populations, however, and the potential sources of more favorable interactions are undescribed. Here, we report one of the first microbiome comparisons of plants from multiple native and invading populations, in the noxious weed yellow starthistle. We identify clear differences in composition and diversity of microbiome bacteria. Our findings raise new questions about the sources of these differences, and we outline the next generation of research that will be required to connect microbiome variation to its potential role in plant invasions.

## Abstract (precis)

Previous studies have found that introduced plants commonly experience more favorable microbial interactions in their non-native range, suggesting that changes to the microbiome could be an important contributor to invasion success. Little is known about microbiome variation across native and invading populations, however, and the potential sources of more favorable interactions are undescribed. Here, we report one of the first microbiome comparisons of plants from multiple native and invading populations, in the noxious weed yellow starthistle. We identify clear differences in composition and diversity of microbiome bacteria. Our findings raise new questions about the sources of these differences, and we outline the next generation of research that will be required to connect microbiome variation to its potential role in plant invasions.

## INTRODUCTION

Humans continue to transport plant species around the globe, and increasing numbers of these translocations result in the invasive expansion of non-native species into recipient communities (1–4). While there are undoubtedly many reasons that species introductions lead to invasions, there is growing evidence that novel species interactions may facilitate the invasive spread of populations (5, 6). Initially, hypotheses about the contribution of species interactions to invasions focused on the potential for non-native species to escape from aboveground herbivores, which are easily observed (7), though it is not clear that herbivore escape is a frequent mechanism of invasion (8–11). More recently, there has been increasing recognition that microbial taxa above- and belowground can have large effects on plant fitness, both positive and negative, and could thus determine whether invasive plants benefit from novel species interactions (5, 10, 12–17). Plant-associated microbial communities have been historically difficult to observe, however, and studies that leverage newly available tools to identify differences in these interactions across native and invading populations are needed to evaluate alternative hypotheses for invasion success (18).

Microbial communities have emerged as particularly likely candidates for facilitating invasions. Although many interactions between plants and microbes can be beneficial, soil microbial communities often appear to have negative net effects on plant fitness which may become more negative over time, e.g., via plant-soil feedbacks (15, 19–21). These interactions between plants and their microbiomes can vary over space and environment (22–25), creating opportunities for introduced plants to escape negative interactions that might characterize their native ranges. Moreover, reductions in microbial diversity can occur in response to environmental change and human disturbances, and lowered microbial diversity could reduce the resistance of ecosystems to invasion (16, 18, 26, 27).

Invasive plant species have provided some of the best evidence to date that microbial interactions can be locally evolved and can vary considerably over geographic regions (28). Introduced plants have been shown to vary in their response to soil communities from their native and invaded ranges, and there are now many examples of more favorable interactions between plants and soil from their invaded range, consistent with escape from enemies or a gain of mutualists during invasion (5, 12, 15, 16, 29–31). Plant-microbe interactions which provide relative benefits to invasive species can be explained by reduced negative effects of key microbial pathogens, increased direct beneficial effects of mutualistic taxa, or increased indirect benefits from taxa that affect competitors more negatively than the invader (18). It is also possible that invaders could benefit from a reduced diversity of enemy interactions, as a result of an associated reduction in ecological costs that derive from simultaneously deploying different defense responses against many different enemies (32–34). These hypotheses all require that there are differences in the microbial communities associated with invading versus native plants; however, the composition of microbial communities associated with different populations of invasive plants remains largely unknown (18).

Here, we conducted one of the first comparisons of plant microbiomes between invading populations and populations in the native source region. We surveyed plant-associated microbial communities in the highly invasive forb yellow starthistle (Centaurea solstitialis). Yellow starthistle is native to a wide region of Eurasia and was introduced from Western Europe to South America in the 1600s and North America in the 1800s as a contaminant of alfalfa seed (35, 36). This herbaceous annual is a colonizer of grassland ecosystems and is often cited as one of the “10 worst weeds of the West” in North America (37). Its extensive invasion of California in the USA (>14 million acres [38]) is particularly well studied, and invading genotypes in this region have evolved to grow larger and produce more flowers than plants in the native range, suggesting a shift in resource allocation that has favored invasiveness (39–41). Previous research has demonstrated that yellow starthistle throughout all of its native and invaded ranges experiences net fitness reductions when grown with its local soil microbial communities (42–44). These studies have also indicated that this negative interaction is weaker (more favorable) in California, raising the possibility that changes in the microbial community have promoted an aggressive invasion.

We sampled microbial communities associated with leaves (phyllosphere and endosphere) and roots (ectorhizosphere and endorhizosphere) of yellow starthistle plants in both the California invasion and native regions in Europe. Previous experiments with fungicide treatments have shown that plant-soil interactions between yellow starthistle and fungi in California are more negative (less favorable) than those in the native range, inconsistent with a role for fungi in beneficial species interactions in this invasion (45). Here, we focus on documenting the variation in bacterial communities, using high-throughput sequencing of ribosomal 16S amplicon sequences to quantify taxonomic composition and diversity of bacteria in yellow starthistle microbiomes. Microbial communities are known to differ among plant compartments (46) and to be influenced by individual plant genotypes (47–49), and so we explicitly tested for differences in the microbiomes of native and invaded range plants relative to the influence of both plant compartment and plant genotype. Our results reveal clear differences in the microbiomes of native and invading plants, including a lower diversity of bacteria associating with the leaves and roots of invaders. We identified several alternative hypotheses for these differences, and we outlined new research directions required to test for their effects on invader success.

## RESULTS

### Sampling and microbiome sequencing.

**FIG 1:**
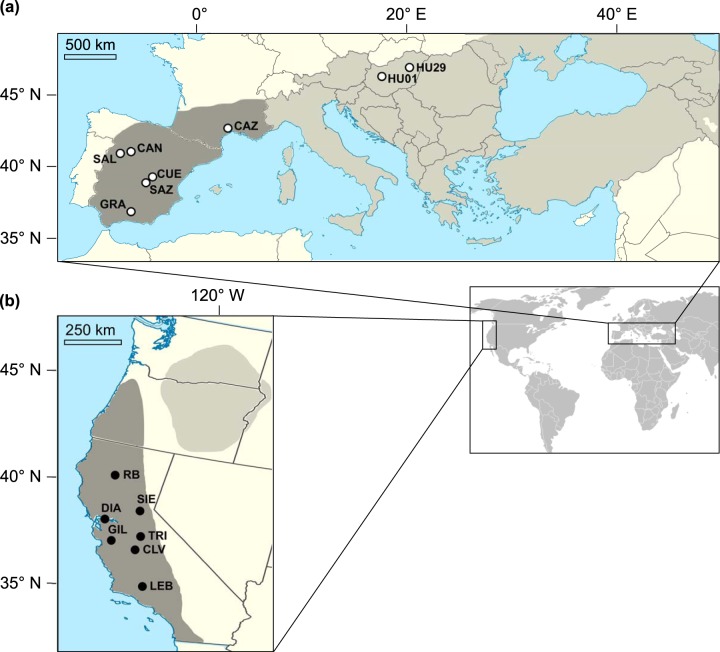
The distribution (gray) of yellow starthistle and sampling sites (circles) for this study. Maps detail the native range in Eurasia (a) and the invasion of western North America (b). Previous work has indicated that Western Europe is the source for the severe invasion in CA, USA (both in dark shading; Barker et al. [36]). Sampling included seven locations in California (b, filled circles), six locations in Western Europe, and an additional two locations in Eastern Europe (a, open circles).

We sampled fifteen populations of yellow starthistle for their microbiomes: seven sampling sites across the invasion of California, six sites in Western Europe (native source region), and two in Eastern Europe (Fig. 1; see also Table S1 in the supplemental material). Tissue was collected from 25 plants per site at 1-m intervals along linear transects. DNA was extracted from surface and endophyte fractions of leaves and roots each pooled by sampling site (15 total populations) and as individual plant samples from 8 plants from each of 10 populations (80 total plants).

10.1128/mSphere.00088-19.6 TABLE S1 Collecting information for populations sampled in this study, including designation codes, localities, coordinates, sampling dates, and specimen accession numbers of plant collections at ARIZ. Download Table S1, DOCX file, 0.1 MB. Copyright © 2019 Lu-Irving et al. 2019 Lu-Irving et al. This content is distributed under the terms of the Creative Commons Attribution 4.0 International license.

To survey bacterial communities, we amplified the V4 region of the 16S rRNA locus. Library preparation utilized peptide nucleic acid clamps (PNAs) to block amplification of plant chloroplast and mitochondrial 16S (50), including a custom chloroplast PNA that we developed to account for sequence divergence in Asteraceae (51). Library preparation followed a dual-index approach (52), and samples were sequenced using 2 × 300-bp paired-end reads on an Illumina MiSeq.

Sequencing yielded 9,672,898 read pairs, of which 6,217,852 remained after merging and quality control; these were 253 bp in length after removing adapter and primer sequences. The numbers of raw read counts per sample ranged from 16 to 306,200 with a median of 21,964. Analysis of the merged and processed reads resulted in 4,014 operational taxonomic units (OTUs), of which 60 were identified as plastid or mitochondrial and 428 were unidentifiable (11%). Of the remaining 3,526, 206 were identified to species (6%), 1,084 to genus (27%), and 2,229 to family (56%) levels. A total of 103 OTUs (3%) were identified as members of the 49 genera with known plant pathogens in the FAPROTAX v.1.1 database (53).

Sequence reads representing yellow starthistle chloroplast and mitochondrial 16S accounted for 40% and 1% of all reads, respectively. Amplification of host chloroplast in samples using the Asteraceae-specific plastid PNA was reduced by up to 51% compared with the Lundberg et al. (50) PNA (see Table S2). Despite PNA blocking activity, 83% of the total reads from leaf endosphere samples were yellow starthistle chloroplast sequences. After the removal of chloroplast and mitochondrial reads, the remaining read counts for most leaf endosphere samples were low relative to the controls (see Fig. S1), and so no further analysis of leaf endosphere bacterial communities was performed.

10.1128/mSphere.00088-19.1 FIG S1 Frequency distributions of unrarefied read counts for samples from all four plant compartments (native and invading population samples combined), as well as control (blank) samples. Vertical red lines indicate thresholds for rarefaction where relevant. Download FIG S1, EPS file, 1.3 MB. Copyright © 2019 Lu-Irving et al. 2019 Lu-Irving et al. This content is distributed under the terms of the Creative Commons Attribution 4.0 International license.

10.1128/mSphere.00088-19.7 TABLE S2 Comparison of host chloroplast blocking levels achieved by different PNA clamps in pooled endorhizosphere samples from two populations. Download Table S2, DOCX file, 0.05 MB. Copyright © 2019 Lu-Irving et al. 2019 Lu-Irving et al. This content is distributed under the terms of the Creative Commons Attribution 4.0 International license.

Rarefaction levels (chosen to reflect the minimum number of reads per sample by compartment, not including outliers) were 18,000 reads per sample for phyllosphere, 17,000 for ectorhizosphere, and 5,000 for endorhizosphere samples (Fig. S1). These levels were also higher than nearly all control samples. Rarefaction cutoffs resulted in the exclusion of five non-control samples which were outliers for low read count: one phyllosphere (code DIA), one ectorhizosphere (code SAZ), and three individual endorhizosphere samples (two from SAZ, one from SIE). A nonmetric multidimensional scaling (NMDS) ordination of all unrarefied samples showed that the controls clustered together and were clearly differentiated from all samples in all plant compartments other than the leaf endosphere (see Fig. S2).

10.1128/mSphere.00088-19.2 FIG S2 NMDS plot of bacterial OTU composition in unrarefied control, phyllosphere, leaf endosphere, ectorhizosphere, and endorhizosphere samples (stress, 0.10). Download FIG S2, EPS file, 0.8 MB. Copyright © 2019 Lu-Irving et al. 2019 Lu-Irving et al. This content is distributed under the terms of the Creative Commons Attribution 4.0 International license.

### Microbiome analyses.

**FIG 2:**
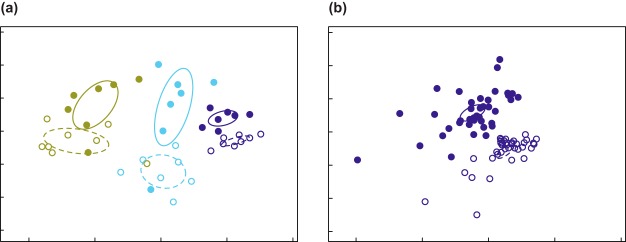
NMDS plots of bacterial OTU composition in phyllosphere (green), ectorhizosphere (light blue), and endorhizosphere (dark blue) samples from native (open symbols) and invaded (closed symbols) ranges. Plotted are pooled samples for each sampling location, showing overall separation by range within compartment (stress, 0.14) (a), and individual plant endorhizosphere samples within native and invading populations (stress, 0.16) (b). Ellipses indicate 95% confidence intervals for samples grouped by range (native range, dashed lines; invaded range, solid lines).

Results from NMDS ordination indicated that bacterial communities differed overall among the phyllosphere, ectorhizosphere, and endorhizosphere compartments (stress, 0.14; *P = *0.001) (Fig. 2a). Within compartments, NMDS further revealed significant differences between native and invaded range endorhizosphere samples (stress, 0.16; *P = *0.001) (Fig. 2b) and ectorhizosphere samples (*P = *0.001). Native and invaded range phyllosphere samples differed with marginal significance (*P = *0.05). Clustering analyses within the phyllosphere and ectorhizosphere compartments consistently grouped invaded range samples together, as well as samples from the source region in Western Europe (see Fig. S3). Native range samples from Eastern Europe (HU01 and HU29) clustered together in these compartments but were variable in their relationship to the other regions. Endorhizosphere samples pooled by location showed less consistent clustering by range.

10.1128/mSphere.00088-19.3 FIG S3 Yellow starthistle populations clustered according to Bray-Curtis dissimilarity between rarefied, square root-transformed pooled samples from three plant compartments. Download FIG S3, EPS file, 0.7 MB. Copyright © 2019 Lu-Irving et al. 2019 Lu-Irving et al. This content is distributed under the terms of the Creative Commons Attribution 4.0 International license.

**FIG 3:**
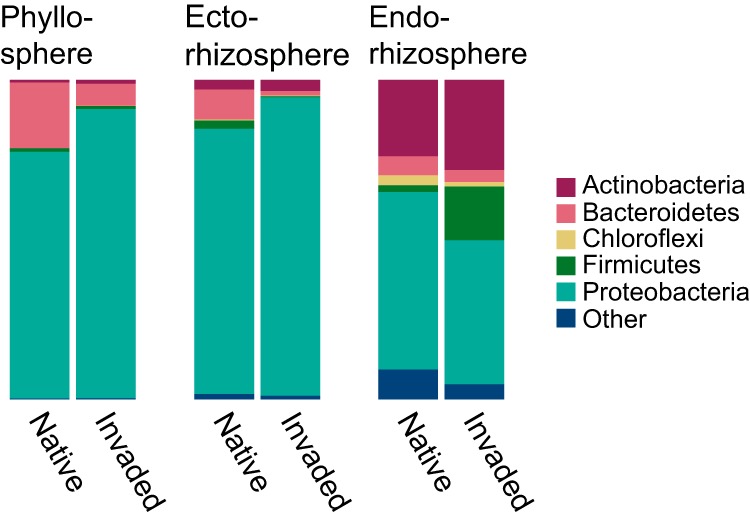
Relative abundances of (proportions of reads mapping to) phyla in yellow starthistle phyllosphere, ectorhizosphere, and endorhizosphere samples from native and invaded ranges.

The dominant phyla among all bacterial communities in both ranges were *Proteobacteria*, *Actinobacteria*, *Bacteroidetes*, and *Firmicutes* (Fig. 3). Principal-component analyses suggested that the strongest contributions to variation in bacterial community composition among populations (within compartments) were made by shifts in the representation of *Bacillus* (*Firmicutes*), *Chryseobacterium* (*Bacteroidetes*), and the *Proteobacteria* taxa *Erwinia*, *Pseudomonas*, and *Xanthomonadaceae* (see Table S3 and Fig. S4). All of these taxa other than *Chryseobacterium* include known plant pathogens in the FAPROTAX v.1.1 database (53).

10.1128/mSphere.00088-19.4 FIG S4 Heatmaps showing the fraction of reads mapping to each of the top 25 OTUs in rarefied bulk samples from three plant compartments. The top five OTUs contributing to the first principal component separating native and invaded samples in each compartment are indicated with an asterisk. Taxonomic assignments for each OTU are given to the lowest taxonomic rank identified. Download FIG S4, EPS file, 0.6 MB. Copyright © 2019 Lu-Irving et al. 2019 Lu-Irving et al. This content is distributed under the terms of the Creative Commons Attribution 4.0 International license.

10.1128/mSphere.00088-19.8 TABLE S3 Loading by top 10 individual OTUs along first principal component axis from analysis of Hellinger-transformed data matrices for three plant compartments. Download Table S3, DOCX file, 0.08 MB. Copyright © 2019 Lu-Irving et al. 2019 Lu-Irving et al. This content is distributed under the terms of the Creative Commons Attribution 4.0 International license.

**FIG 4:**
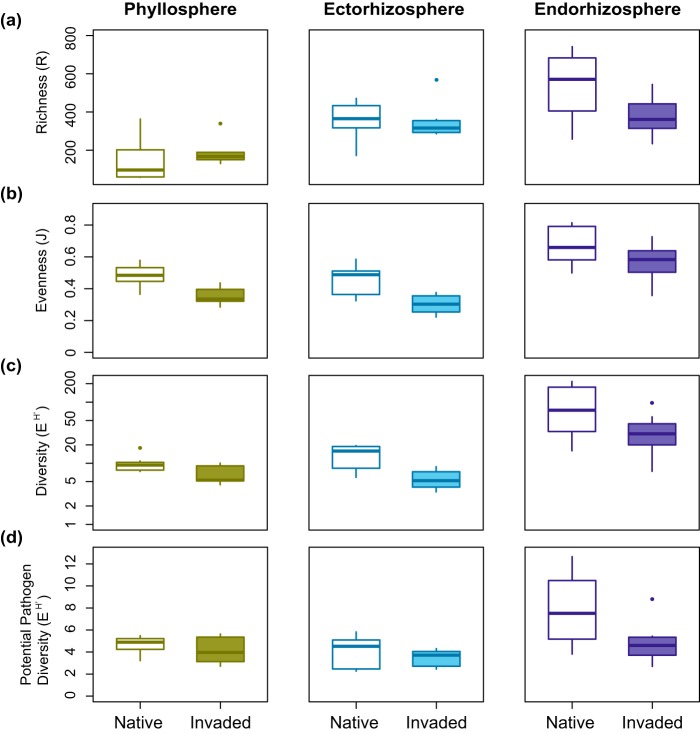
Distributions of OTU richness (a), evenness (b), and diversity (e^H′^) (c and d) among samples (pooled plants) from each location in the native and invaded ranges for phyllosphere, ectorhizosphere, and endorhizosphere compartments. Panels a to c show values for all OTUs and panel d shows values based on OTUs from known pathogen-containing genera.

**FIG 5:**
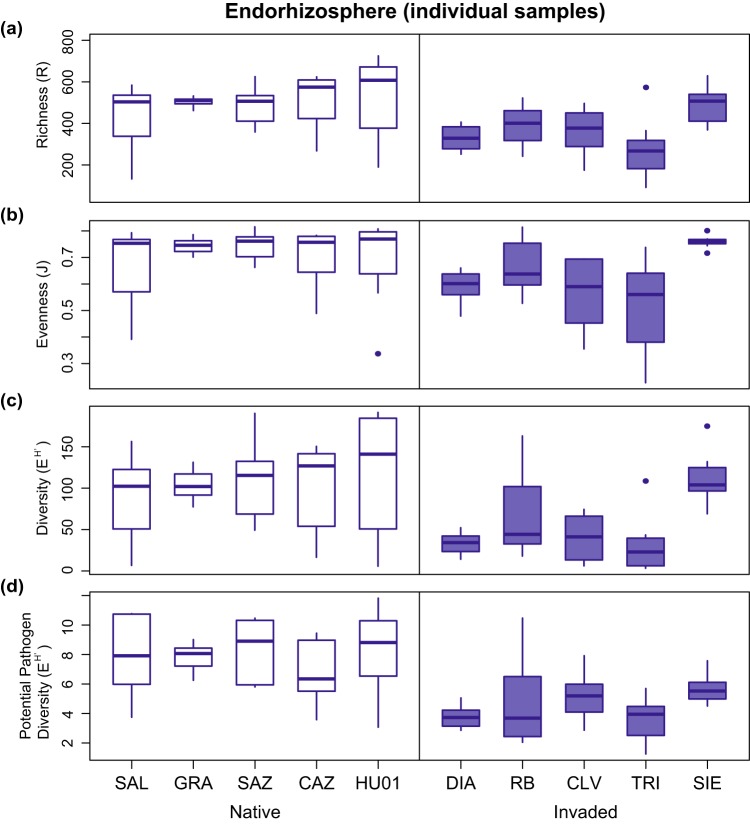
Distributions of endorhizosphere OTU richness (a), evenness (b), and diversity (e^H′^) (c and d) among individual plants at each location in the native and invaded ranges. Panels a to c show values for all OTUs and panel d shows values based on OTUs from known pathogen-containing genera.

In general, bacterial OTUs showed a pattern of lower median richness (R), evenness (J), and diversity (e^H′^) for plants from invaded range sites in all compartments, with the exception of richness in the phyllosphere (Fig. 4 and 5). Within the phyllosphere, invaders were not significantly different in richness ($\chi_{1}^{2}$ = 1.67, *P = *0.20) but were significantly lower in evenness ($\chi_{1}^{2}$ = 8.07, *P = *0.005) and were marginally lower in Hill diversity ($\chi_{1}^{2}$ = 3.75, *P = *0.05) than native range plants. Similarly, the ectorhizosphere of invaders was not significantly different in richness ($\chi_{1}^{2}$ = 0.69, *P = *0.41) but was significantly lower in both evenness ($\chi_{1}^{2}$ = 5.0, *P = *0.03) and diversity ($\chi_{1}^{2}$ = 6.21, *P = *0.01). In contrast, endorhizosphere samples pooled by site did not differ significantly in evenness ($\chi_{1}^{2}$ = 2.26, *P = *0.13) but were marginally significantly lower in richness ($\chi_{1}^{2}$ = 3.43, *P = *0.06) and diversity ($\chi_{1}^{2}$ = 3.01, *P = *0.08). Nested analysis of variance (ANOVA) of individual endorhizosphere samples indicated strongly significant reductions in richness, evenness, and diversity in invading plants (fixed effect of region, all *P < *0.001) (Fig. 5). For individual endorhizosphere samples, populations did not differ significantly in any metrics within native/invaded regions, with the exception of significantly higher evenness in plants at site SIE relative to site TRI in the invaded range.

To explore the potential for these patterns to capture plant-pathogen interaction in particular, we filtered the rarefied data sets for genera containing known plant pathogens in the FAPROTAX database, which resulted in 49 phyllosphere OTUs, 69 ectorhizosphere OTUs, and 88 endorhizosphere OTUs. While pathogen-containing genera are likely to include both pathogenic and nonpathogenic strains (54), we expected that our pathogen-containing OTU data set would be enriched for potential plant pathogens, relative to the full data set. *Pseudomonas* and *Erwinia* were among the most common pathogen-containing genera encountered in each plant compartment in both ranges. The phyllosphere also included a high frequency of *Janthinobacterium*, with a relative increase in *Serratia* in the invaded range. In the ectorhizosphere, *Serratia* was common in both ranges, but invading plants showed a large relative decline in *Erwinia* and increase in *Pseudomonas*. In the endorhizosphere, invading plants harbored less *Pseudomonas* and more *Bacillus* and *Streptomyces* than native plants (see Fig. S5). The diversity of these OTUs showed similar trends to the total diversity, with lower median values in invaded range root compartments. No differences between regions were statistically significant for the phyllosphere ($\chi_{1}^{2}$ = 0.60, *P = *0.44) or ectorhizosphere ($\chi_{1}^{2}$ = 0.49, *P = *0.48). For the endorhizosphere samples pooled by site, significantly lower diversity was indicated in the invaded range ($\chi_{1}^{2}$ = 4.34, *P = *0.04). For individual endorhizosphere samples, nested ANOVA also indicated significantly lower diversity in the invaded range (*P < *0.0001), and no significant differences among populations within regions.

10.1128/mSphere.00088-19.5 FIG S5 Proportion of rarefied read counts assigned to pathogen-containing genera in all samples according to plant compartment and range. Download FIG S5, EPS file, 1.2 MB. Copyright © 2019 Lu-Irving et al. 2019 Lu-Irving et al. This content is distributed under the terms of the Creative Commons Attribution 4.0 International license.

**FIG 6:**
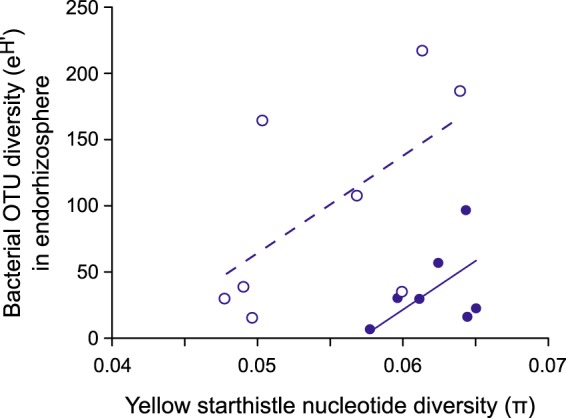
Bacterial diversity (e^H′^) in endorhizosphere samples pooled by sampling location, as a function of the genetic diversity among plants at the same sites (calculated as the average proportion of pairwise nucleotide differences between alleles [π] at variable positions in the genome; from Barker et al. [36]). Lines show significant positive relationships (linear model, *P = *0.02) between microbial and plant diversity at sampling locations in both the native range (open symbols, dashed line) and the invaded range (closed symbols, solid line).

Finally, we examined the influence of plant genotype on microbial composition. Our geographic regions correspond to genetically differentiated subpopulations, and within these regions, our study sites are also known to vary in plant genetic diversity (36). Using estimates of the average proportion of pairwise nucleotide differences between alleles (π) for plants at each site from (36), we predicted microbial diversity estimates (e^H′^) for each plant compartment using linear models with fixed effects of plant genetic diversity, region (native versus invaded), and the interaction between these two effects. The model was significant for endorhizosphere samples pooled by site [*F*_(2,12)_ = 5.89; *P = *0.02; $r_{\text{adj}}^{2}$ = 0.41) (Fig. 6), with significant main effects of both plant genetic diversity (*P = *0.03) and region (native versus invaded, *P = *0.006). The interaction between these two effects was not significant (*P = *0.71) and was removed from the final model. This same pattern was marginally significant when using only OTUs from pathogen-containing genera in the endorhizosphere samples (effect of plant genetic diversity, *P = *0.08). Similar linear models did not identify significant effects of plant genetic diversity when predicting the median diversity of individual plant endorhizosphere samples (*P = *0.74) or diversity in the phyllosphere (*P = *0.35). There was a marginally significant positive effect of plant genetic diversity on diversity in the ectorhizosphere (*P = *0.08) in addition to the effect of region (*P = *0.002).

## DISCUSSION

Introduced plants will encounter a variety of novel species interactions as they establish across biogeographic regions. For many plants, severe invasions are associated with more favorable interactions with soil microbial communities (18, 44). We found that bacterial microbiomes of invading yellow starthistle were unique in composition and lower in diversity relative to the bacterial microbiomes of plants from the native range, differences that persisted within plant compartments and across variation in plant genetic diversity.

As observed in other species, bacterial communities differed most among plant compartments (46, 55). The numbers and diversity of taxa within each compartment were similar in magnitude to those reported in other studies of prokaryotic 16S sequences, e.g., from Agavaceae (56), Brassicaceae (57), Cactaceae (58), and other Asteraceae (59). The dominant phyla were *Proteobacteria*, *Actinobacteria*, *Bacteroidetes*, and *Firmicutes*, which are also characteristic of plant-associated bacterial communities surveyed to date (46). The exception was the leaf endosphere, where a paucity of sequences relative to controls suggests that persistent chloroplast contamination obscured low frequency endophytes, despite our development of an Asteraceae-specific PNA (51). A targeted survey is needed to better characterize this compartment (e.g., quantitative PCR [60]).

Notably, diversity was approximately twice as high in the endorhizosphere as in the ectorhizosphere. Current reviews have concluded that root endosphere communities are typically less diverse than those in the ectorhizosphere (46, 55). Our root collections were washed but not surface sterilized and may represent some of the rhizoplane/rhizosphere in addition to the endosphere, elevating our estimates of diversity. It is also possible that yellow starthistle deviates from initially reported patterns, which have also been challenged by other recent studies (58, 59).

Within compartments, the community composition was consistently different between samples from native and invaded ranges and included shifts in taxa across all major groups. Our native range samples represented a larger geographic area and spanned distinct genetic subpopulations of yellow starthistle, but native range sites clustered together in overall community composition and there was little evidence of individual site differences within ranges. Between ranges, the diversity of OTUs was lower in the invaded range, a pattern that was dominated by lower evenness of OTUs in both the phyllosphere and ectorhizosphere and by lower richness of OTUs in the endorhizosphere. Thus, invading plants were more strongly dominated by a few taxa at high relative abundance on root and leaf surfaces and harbored fewer bacterial taxa in their root endophytic communities.

We observed a significant positive association between root microbial diversity and genotypic diversity among plants at the population scale. This association was strongest in the endorhizosphere, the only endophytic compartment in our analysis, consistent with plant genotype having the largest influence on microbial taxa colonizing within the plant itself (49, 61, 62). Within-species plant genotype effects have been observed previously and may interact with the effect of environment to shape microbial communities (24, 47). Interactions between plant and microbial diversity could be particularly important for invasive species, where genetic bottlenecks during establishment and range expansion can reduce genetic diversity among plants (63–65). Nevertheless, it appears that genotype effects are often minor relative to site effects (47–49, 66), and we found that genotypic effects were evident only within regions and did not explain microbial diversity differences between regions.

We propose three potential explanations for the regional differences in microbiome diversity that we have observed, which are not mutually exclusive. First, the regional microbial environment might be less diverse in western North America in general, such that plant microbiomes simply reflect the diversity present in their external environment. Second, yellow starthistle invasions might cause reductions in bacterial diversity in the environment. Third, invading yellow starthistle might be experiencing an outbreak of microbial infection, resulting in high abundance of a few taxa and reduced overall diversity in our samples. We discuss each of these hypotheses in turn below.

Microbial environments outside plants (e.g., in the soil and air) are known to vary at continental scales (47, 57, 67). A variety of factors may explain this geographic variation, particularly abiotic differences (22, 46, 55, 68). Soil type appears to have a particularly strong influence on microbial communities (e.g., see references 49 and 61), and is known to differ broadly across yellow starthistle’s range (45). In addition, populations in California are at the warm and dry extreme of yellow starthistle’s climatic niche (41), and our sampling was conducted at the end of a period of severe drought (69, 70), which could have amplified any microbial differences related to climate (71). Interestingly, a recent study of grassland plants found that microbial diversity increased under drought, whereas we found reduced diversity in the drought-affected range (72). A critical comparison of yellow starthistle’s external microbial environment in each range should include broad spatial and temporal sampling of soil and atmospheric microbial communities from which its microbiomes are assembling (73–75). A major challenge will be disentangling any regional differences in such samples from the effects of yellow starthistle itself, as we detail below.

Importantly, yellow starthistle’s invasion might also cause changes to its microbial environment. Species invasions have been shown to alter microbial composition over short timescales (76–78), though long-term effects are less clear (79, 80). Yellow starthistle invasions are denser than populations in the native range by an order of magnitude or more (42, 81) and include a lower diversity of plant species overall (82–84). Low plant diversity can sometimes depress the diversity of microbes in the environment and within plants (26, 56, 85). This means that invasions could be a cause rather than a result of observed weaker plant-soil interactions for invasive species in their introduced ranges (15, 18). Ideally, microbial communities would be compared among regions using samples outside patches of invasive plants, in a suitable habitat that has not yet been affected by an invasion, though identifying suitable but uninvaded habitat for these comparisons is not trivial. More practically, observing the development of soil microbial communities during plant-soil feedback experiments should be informative regarding the influence of invaders on microbial environments (18).

Finally, our invading plant populations might be experiencing disease outbreaks, resulting in a high abundance of a few strains and lower diversity of the microbiota as a whole. We think this is less likely, however. Disease outbreaks in plants tend to be local in scale, with microbial communities varying over the spatial scale of meters (86). Our invading populations are widely separated (e.g., they span >5° latitude), such that broadly shared disease patterns would imply that outbreaks are more common in the invading populations overall. Yet, it has been argued that invaders are successful precisely because they experience lower levels of attack from pathogens and other enemies (13, 15, 16), and yellow starthistle specifically has been shown to have more favorable interactions with its invaded range soil community (44). Again, broad spatial and temporal microbial sampling would be informative here for identifying outbreak dynamics, and plant-microbe interaction experiments in culture or field plantings would help to resolve the fitness effects of dominant strains.

To date, few studies have compared microbial community composition between the native and introduced ranges of invasive plants. McGinn and colleagues (87) reported no differences in the diversity of mutualistic fungal taxa associated with the roots of multiple species of European *Trifolium* introduced to New Zealand, despite more favorable soil interactions in the invasions (88; but see reference 89). Johansen and colleagues (90) found increased diversity of fungal communities on the roots of European Ammophila arenaria invading Australia and New Zealand, though there appear to be no differences in interactions with soil microbial communities in its invasions (in North America [91]). Gundale and colleagues explored the potential contribution of fungal endophyte communities to more favorable (negative) soil interactions observed in introduced plantations of lodgepole pine (Pinus contorta) from North America (92, 93). For lodgepole pine, microbial communities differed among several global regions examined, but there was no consistent pattern of loss of potential fungal pathogens or gain of mutualists in the introductions, and it remains unclear what part of the soil community is responsible for the observed differences in interactions across ranges (92). Reinhart and colleagues (94) focused specifically on *Pythium* fungal pathogens and quantified their virulence on North American Prunus serotina introduced to Europe. They found that the most virulent strains occurred only in the native range, consistent with benefits to invading plants escaping this specific pathogenic group. In the only microbiome comparison that included bacterial taxa, Finkel and colleagues (95, 96) explored the phyllosphere community of multiple species of *Tamarix* in native and introduced parts of their ranges, finding that microbial communities were in general most strongly structured by geographic region.

Our study is the first to find consistent differences in the microbiomes of native and invading plants which coincide with documented fitness differences in plant interactions with soil communities (44). Among the few invader microbiome studies to date, ours is unusual in focusing on the bacterial community. Fungi have historically received more attention for their fitness effects on plants, but bacteria can also play a critical role both as pathogens and mutualists (55, 97, 98). For yellow starthistle, previous experiments have demonstrated that fungal communities are not responsible for more favorable conditions in the invaded range (45), and our findings indicate that bacterial communities warrant further investigation as the potential source of these differences. The fitness effects of our specific OTUs are unknown, however, and identifying the bacterial OTUs that accumulate during interactions with plants would help to elucidate important pathogenic or mutualistic taxa and allow field surveys to explicitly test hypotheses that these strains are lost or gained in the invaded environment.

We have previously argued that yellow starthistle has benefitted from the historical loss of plant competitors in California (41). Disturbance is critical for yellow starthistle establishment, and functionally similar native species compete well against it in experiments; however, key competitors have been lost from the ecosystem due to perturbations prior to yellow starthistle invasion (45, 83, 99–101). Any benefits of altered bacterial communities could be independent of competition with native plant species, but these factors might also interact. Increased density due to a lack of competition could have reduced plant-associated microbial diversity, as described above. Yellow starthistle experiences negative plant-soil feedbacks across generations (42), however, and the build-up of high plant densities is therefore unlikely to generate more favorable soil interactions. Alternatively, the historical loss of native species diversity in California (84) could have resulted in the loss of associated microbial diversity (26, 56, 85), generating particularly strong opportunities for invasion into a system with both reduced plant competition and reduced pathogen diversity. Microbial surveys of remnant native communities, as well as across densities of yellow starthistle, would facilitate tests of alternative hypotheses for interacting effects of plant and microbial diversity, and it may be informative to explore microbial communities preserved on native plant specimens predating the extensive invasion of yellow starthistle in this region.

In conclusion, we found consistent differences between native and invading yellow starthistle plants in their bacterial microbiomes. These differences were robust to additional variation associated with plant compartment and the diversity of plant genotypes. Invaded range microbiomes differed in composition across major taxonomic groups and harbored a lower diversity of bacteria, including reduced evenness on the surfaces of leaves and roots and reduced richness of root endophytes. We suggest that bacteria could be the source of more favorable microbial interactions that have been previously observed in this invasion. Our findings also raise questions about (i) whether lower bacterial diversity is a feature of the invaded environment or whether it is caused by the invasion itself, and (ii) how specific differences in the microbial community affect plant fitness. These questions highlight the need for additional studies that compare microbial communities (including bacteria) associated with native and invading populations, that couple microbial community identification with plant-soil feedback and fitness experiments, and that examine the interaction of environment, plant diversity, and plant density on microbial communities and their fitness effects on plants.

## MATERIALS AND METHODS

### Study species.

Yellow starthistle (Centaurea solstitialis L., Asteraceae) is an obligately outcrossing annual plant, diploid throughout its range (102). Plants form a taproot and grow as a rosette through mild winter and/or spring conditions, bolting and producing flowering heads (capitula) throughout the summer. The species is native to Eurasia, where distinct genetic subpopulations have been identified in Mediterranean Western Europe, central-eastern Europe, Asia (including the Middle East), and the Balkan-Apennine peninsulas (36). The invasion in California, as well as invasions in South America, appears to be derived almost entirely from Western European genotypes (Fig. 1) (36).

### Sample collection.

At each location in June-July 2015, plants were sampled every meter (or to the nearest meter mark) along a 25-m transect, to yield microbial samples from 25 individual plants per population. Individuals in rosette or early bolting stages were preferentially selected. In one population (HU29), low plant density yielded 20 individuals along the 25-m transect. Using sterile technique, plants were manually pulled and each individual sampled using modified versions of protocols by Lundberg et al. (49) and Lebeis et al. (62) as described below. Plants were pressed and dried after sampling and submitted to the University of Arizona Herbarium (ARIZ) (see Table S1 in the supplemental material).

### (i) Phyllosphere and ectorhizosphere.

One to three basal nonsenescent leaves were collected from each plant, as well as the upper 2 to 5 cm of the taproot, together with accompanying lateral roots (excess soil was brushed or shaken off). Leaf and root samples were placed in individual 50-ml tubes containing 25 ml of sterile wash solution (45.9 mM NaH_2_PO_4_, 61.6 mM Na_2_HPO_4_, 0.1% Tween 20). Tubes were shaken by hand for 1 min (timed). Leaf and root samples were then removed and stored on ice in separate tubes (leaves in empty tubes, roots in tubes containing 10 ml of wash solution) until further processing. Wash samples were stored on ice during transport and then refrigerated at 4°C. Phyllosphere and ectorhizosphere washes were pooled across all (20 or 25) plants at a location and then centrifuged at 2,200 × g at 4°C for 15 min. Supernatants were discarded, and pellets were air-dried and stored at −20°C until DNA extraction.

### (ii) Leaf endosphere.

Leaves were surface sterilized by submerging in bleach solution (10% commercial bleach, 0.1% Tween 20) for 2 min. Leaves were then rinsed in distilled water, patted dry using a Kimwipe, and sealed in individual sterile surgical envelopes (Fisherbrand 01-812-50). Envelopes were kept in silica gel desiccant until leaf tissue was completely dry and then stored at room temperature until DNA extraction.

### (iii) Endorhizosphere.

Roots were further washed by shaking in 10 ml of wash solution until visible residual soil was removed. Washed roots were stored and dried as described above for leaves.

### (iv) Controls.

At each collection site, a tube of sterile wash solution was left uncapped while sampling plants. Disinfected tools were periodically agitated in the blank wash tube before sterilization and use for the next sample collection. For each population, rinse water and wipes used to process tissue samples were represented in controls by rinsing and wiping flame-sterilized forceps and then agitating the forceps in the blank wash tube. Controls were stored and processed in the same manner as for phyllosphere and ectorhizosphere samples.

### DNA extraction.

Extractions were carried out using sterile technique in a laminar flow hood. For pooled tissue extractions, equal sections of leaf tissue (50 mm^2^) and root tissue (12.5 mm^3^ plus 10 mm of lateral roots) were collected from each individual sample per location and pooled prior to extraction. Control (blank) samples were collected for each batch of extractions by swabbing tools and surfaces and then extracting DNA from the swab head.

All DNA samples were extracted using the Mo Bio PowerSoil kit (Mo Bio Laboratories, Inc.). Phyllosphere and ectorhizosphere DNA was extracted from up to 0.25 g of wash pellets according to the standard kit protocol. Leaf and root tissues were ground to powder or sawdust consistency in liquid nitrogen using sterile mortars and pestles. Leaf and root DNA was extracted from 20 mg (leaf) or 100 mg (root) of ground tissue with the following modification to the standard protocol: tissue was incubated at 65°C for 10 min in extraction buffer and then vortexed for 1 min, followed by a second 10-min incubation (as described under “alternative lysis methods” in the kit protocol). Control DNA was extracted by placing whole swab heads directly into extraction tubes. Extracted DNA was eluted in PCR-grade water and stored at −20°C pending library preparation.

### Library preparation and sequencing.

To remove secondary compounds inhibiting PCR, DNA extracted from root and leaf tissue (together with corresponding blanks) was purified using a ZR-96 genomic DNA cleanup kit (Zymo Research). All DNA concentrations were quantified using a Qubit fluorometer high-sensitivity assay for double-stranded DNA (Invitrogen) and standardized to equimolar amounts.

Library preparation followed a dual-index approach (52) using a two-step PCR protocol as follows. In the first step (target-specific PCR), the V4 region of the 16S rRNA gene was amplified using target-specific primers (515F and 806R; 103) appended with common sequence (CS) tags through a linker sequence which varied from two to five nucleotides in length. Target-specific PCR was carried out using Phusion Flash master mix (Thermo Scientific) in a 25-μl reaction mixture volume in a Mastercycler Pro thermocycler (Eppendorf) under the following conditions: 25 cycles of 1 s at 98°C, 5 s at 78°C, 5 s at 57°C, and 15 s at 72°C. Products were visualized on an agarose gel and diluted up to 1:15 (depending on yield); 1 μl of diluted product was then used as the template in the second step (barcode-adapter attachment PCR). Using reagents and equipment as described above, barcoded primer pairs incorporating Illumina P5 and P7 adapters were used to amplify products from target-specific PCR in 25-μl reaction mixture volumes under the following conditions: 10 cycles of 1 s at 98°C, 5 s at 78°C, 5 s at 51°C, and 15 s at 72°C. Barcoded amplicons were quantified by fluorometry, pooled in equimolar amounts, cleaned, and submitted to the University of Idaho’s IBEST Genomic Resources Core for quality control (QC) and sequencing. Amplicons were multiplexed to use half the capacity of one 2 × 300-bp run on an Illumina MiSeq platform.

Peptide nucleic acid clamps (PNAs) were included in both PCR steps of library preparation to block amplification of plant chloroplast and mitochondrial 16S as recommended by Lundberg et al. (50). Clamp sequences published by Lundberg et al. (50) were compared with chloroplast and mitochondrial 16S sequences from yellow starthistle and three other species of Asteraceae with published organellar genomes (Centaurea diffusa, Helianthus annuus, and Lactuca sativa). We found a single nucleotide mismatch between the Asteraceae chloroplast 16S and the plastid PNA sequences, and designed an alternative plastid PNA specific to the Asteraceae sequence (5′-GGCTCAACTCTGGACAG-3′) (51). All samples for this study were amplified using the plastid PNA of our design, together with the mitochondrial PNA published by Lundberg et al. (50). To gauge the effectiveness of our alternative PNA, two duplicate samples were processed using both PNAs published by Lundberg et al. (50).

### Identification of operational taxa and potential plant pathogens.

Demultiplexed paired reads were merged and quality filtered using tools from the USEARCH package version 9.0.2132 (104). Merged reads were truncated to uniform lengths, and primer sequences were removed using a combination of the seqtk toolkit version 1.2 (https://github.com/lh3/seqtk) and a custom script. The UPARSE pipeline (105) implemented in the USEARCH package was used for further data processing and analysis: unique sequences were identified, and those represented only once or twice in the processed read set were discarded as likely PCR or sequencing errors. Remaining sequences were clustered into operational taxonomic units (OTUs) at a 97% threshold, chimeras were filtered out, and per-sample OTU read counts were tabulated using the UPARSE-OTU algorithm. Assignment of OTUs to nearest taxonomic match in the Greengenes database (106) was carried out using the UCLUST algorithm implemented in QIIME version 1.9.1 (107, 108). Data were further processed using tools from the QIIME package: reads mapping to chloroplast and mitochondrial OTUs were removed, and samples were rarefied by plant compartment. Rarefaction levels were chosen to reflect the distribution of read counts per sample within plant compartments, subsampling to the minimum number of reads necessary to include all samples except those that were outliers for low read count.

Taxa known to contain plant pathogens were identified using the FAPROTAX database (version 1.1 (53). A list of all genera included under the “plant pathogen” functional category was used to filter our OTU tables by taxonomic assignment.

### Microbial community analyses.

All statistical analyses were performed in R (109). We evaluated differences in bacterial community composition between plant compartments and between native and invaded ranges within plant compartments by performing nonmetric multidimensional scaling (NMDS) using the R packages vegan (110) and MASS (111). Individual plant samples or samples pooled within sampling site provided replicates in these comparisons. Ordinations were based on Bray-Curtis distances and were performed using a two-dimensional configuration to minimize stress, using Wisconsin double-standardized and square root-transformed data, with expanded weighted averages of species scores added to the final NMDS solution. Significant differences among plant compartments and between native and invaded samples were assessed using the envfit function in vegan. Ellipses were drawn on NMDS plots using the vegan function ordiellipse, representing 95% confidence limits of the standard error of the weighted average of scores.

We further explored the underlying correlates of bacterial community variation using principal-component analysis (PCA; using R function prcomp) for samples from native and invaded ranges within each plant compartment. Prior to performing the PCA, we performed Hellinger’s transformation to minimize the influence of OTUs with low counts or many zeros (112–114). We then identified the OTUs with the highest loading on the dominant PC axis of variation by examining the matrix of variable loadings produced by prcomp. The OTU composition of samples pooled by sampling site (phyllosphere, ectorhizosphere, and endorhizosphere samples [“pooled samples”]) was visualized using a heatmap generated in ggplot2 (115), and samples were hierarchically clustered by Bray-Curtis dissimilarity (hclust function in R) using McQuitty’s method (116).

We compared the diversity of OTUs between the native and invaded range for each plant compartment using richness (R), evenness (Pielou’s J [117]), and their combined effects via the Hill series exponent e^H′^ (118) of the Shannon diversity index (H′ [119]). Again, individual plant samples or samples pooled by sampling site provided replicates in these comparisons. Diversity values were calculated using the packages vegan and iNEXT (120) and compared between native and invaded ranges using a nonparametric Kruskal-Wallis rank sum test on rarefied read counts. For plant tissue samples that included multiple individuals per site, we compared diversity between regions using a nested ANOVA with fixed effects of region and population nested within region, and among sites using a *post hoc* Kruskal-Wallis test within regions. We conducted these comparisons on a data set including all OTUs and a reduced data set including only OTUs assigned to genera with known plant pathogens according to the FAPROTAX database, as described above.

### Accession numbers.

Sequence data are available under NCBI SRA accession number PRJNA494717. Sampling locations and herbarium accession numbers of plant specimens are given in Table S1.
