# Supplementary material for: Native and Invading Yellow Starthistle (Centaurea solstitialis) Microbiomes Differ in Composition and Diversity of Bacteria
Source: mSphere. 2019 Mar 6;4(2):e00088-19. doi: 10.1128/mSphere.00088-19 (PMC6403453; doi:10.1128/mSphere.00088-19)
Supplement: TABLE S1 [file mSphere.00088-19-st001.docx]

| **Code** | **Lat.** | **Long.** | **Locality** | **Date** | **ARIZ specimen numbers** |
| --- | --- | --- | --- | --- | --- |
| SAL | N40.99112 | W005.65831 | Avenida de la Merced, ~1.5km N of Salamanca. Dirt path between road and field, on E side of road. | 22-Jun-15 | 426137, 426032, 425636, 426125, 426034, 426033, 426035, 426126, 426140, 426068, 426127, 426128, 426129, 426130, 426131, 426132, 426133, 426134, 426135, 426136, 426138, 425635, 426139 |
| CAN | N41.00085 | W004.89715 | Junction AV800 & road to Canales. On E side of Canales road, in the narrow strip between road and field edge. | 22-Jun-15 | 425643, 426065, 426064, 426063, 426055, 426054, 425646, 426067, 426053, 426052, 425642, 426066, 426051, 425641, 426062, 426061, 426059, 426058, 426060, 426057, 426056 |
| GRA | N37.26843 | W003.66488 | GR300 ~100m from junction w 213, ~20m up dirt path to the E side of the road. Weedy waste area near edge of olive orchard. | 23-Jun-15 | 425716, 425715, 425714, 425713, 425739, 425738, 425737, 425736, 425735, 425734, 425733, 425732, 425728, 425740, 425729, 425711, 425702, 425703, 425704, 425705, 425706, 425707, 425708, 425709, 425710 |
| SAZ | N39.83529 | W002.50997 | Gravel road by W side of CM3118 (road to Villares del Saz), just next to underpass under A3. Dirt farming path (for tractors) on S side of road, between sunflower fields. | 24-Jun-15 | 425678, 425741, 425679, 425731, 425680, 425681, 425682, 425683, 425684, 425685, 425757, 425755, 425753, 425751, 425749, 425746, 425744, 425743, 425758, 425756, 425752, 425750, 425748, 425747, 425745, 425742 |
| CUE | N40.12939 | W002.13880 | CM2105 between Cuenca & Tragacete ~8km W of Cuenca. Gravel road to the N of 2105, between field and river. | 24-Jun-15 | 425424, 425421, 425420, 425423, 425425, 425426 |
| CAZ | N43.74958 | E003.77119 | D113, ~1km from junction w 986, near Cazevieille. Flat, gravelly turnout & intersection w unpaved road, & gravelly roadsides. | 20-Jun-15 | 425691, 425690, 425971, 425688, 425689, 425972, 425692, 425970, 425693 |
| HU01 | N47.18240 | E18.08928 | Unnamed dirt roads about 200m NW of E66/8, running parallel. Access by perpendicular unnamed paved road. | 7-Jul-15 | 425655, 425660, 425661, 425653, 425652, 425658, 425657, 425656, 425654, 425631, 425687, 425634, 425633, 425686, 425630, 425628, 425629, 425627, 425662 |
| HU29 | N47.31785 | E21.03385 | Margin of irrigation ditch bordered by hay and sunflower fields on one side and service road parallel to main road (E60/4) on the other side. Site to the south of E60/4. | 8-Jul-15 | 425730, 425677, 425676, 425640, 425675, 425674, 425673, 425726, 425725, 425724, 425723, 425722, 425721, 425720, 425719, 425718, 425717, 425727 |
| DIA | N37.86275 | W121.98003 | Hillside to the West of parking lot at the end of Green Valley Road. | 8-Jun-15 | 425394, 425400, 425401, 425402, 425403, 425404, 425405, 425406, 425407, 425408, 425409, 425115, 425381, 425410, 425382, 425383, 425384, 425385, 425386, 425387, 425388, 425389, 425390, 425391, 425392 |
| GIL | N37.03389 | W121.53611 | Disturbed grassy area adjacent to turn out on South side of Leavesley Rd. near intersection with New Ave. Grassy patch between road and tilled field. | 7-Jun-15 | 425374, 425373, 425380, 425379, 425378, 425377, 425376, 425375, 425124, 425123, 425122, 425121, 425119, 425120, 425118, 425114, 425113, 425399, 425112, 425395, 425111, 425398, 425397, 425396 |
| RB | N40.27085 | W122.27103 | Lightly grassy patch to north-west of rest stop (Herbert S. Miles rest area) between Cottonwood and Red Bluff on southbound I-5. | 9-Jun-15 | 425372, 425371, 425370, 425369, 425368, 425418, 425365, 425415, 425367, 425366, 425416, 425417, 425419 |
| CLV | N36.91603 | W119.79341 | Gully on eastern side of the Yosemite fwy (41) adjacent to olive orchards. | 6-Jun-15 | 426046, 426045, 426044, 426043, 426042, 426036, 426037, 425637, 426038, 426039, 426040, 426041, 425639, 426026, 426027, 426028, 426029, 426030, 426031, 425638 |
| LEB | N34.82844 | W118.87369 | Cal trans maintained, weedy area adjacent to SW of rest stop off of south bound I-5. | 5-Jun-15 | 425968, 425695, 425969, 425966, 425967, 426047, 425694, 426050, 426049, 426048 |
| TRI | N37.46159 | W119.79399 | Strip of grassland along south side of Hwy 49 at intersection with Triangle road. Strip between road and fenced off rangeland. | 11-Jun-15 | 425116, 425117, 425414, 425412, 425413, 425411, 425393 |
| SIE | N38.78162 | W120.41639 | Site upslope of Weber Mill rd. about ¼ mile from the turnoff from Ice House road. | 10-Jun-15 | 425648, 425647, 425649, 425644, 425645 |
