# Supplementary material for: Native and Invading Yellow Starthistle (Centaurea solstitialis) Microbiomes Differ in Composition and Diversity of Bacteria
Source: mSphere. 2019 Mar 6;4(2):e00088-19. doi: 10.1128/mSphere.00088-19 (PMC6403453; doi:10.1128/mSphere.00088-19)
Supplement: TABLE S2 [file mSphere.00088-19-st002.docx]

| **Sample** | **plastid PNA** | **total** | **chloroplast** | **% chloroplast** |
| --- | --- | --- | --- | --- |
| CAN pooled endorhizosphere | Lundberg *et al.* 2013  (GGCTCAACCCTGGACAG) | 14,763 | 2,873 | 19 |
| CAN pooled endorhizosphere | Asteraceae-specific  (GGCTCAACTCTGGACAG) | 12,579 | 91 | 1 |
| CUE pooled endorhizosphere | Lundberg *et al.* 2013  (GGCTCAACCCTGGACAG) | 11,962 | 6,318 | 53 |
| CUE pooled endorhizosphere | Asteraceae-specific  (GGCTCAACTCTGGACAG) | 14,608 | 314 | 2 |
