# Supplementary material for: Native and Invading Yellow Starthistle (Centaurea solstitialis) Microbiomes Differ in Composition and Diversity of Bacteria
Source: mSphere. 2019 Mar 6;4(2):e00088-19. doi: 10.1128/mSphere.00088-19 (PMC6403453; doi:10.1128/mSphere.00088-19)
Supplement: TABLE S3 [file mSphere.00088-19-st003.docx]

| **Phyllosphere** | | |  | **Ectorhizosphere** | | |  | **Endorhizosphere** | | |
| --- | --- | --- | --- | --- | --- | --- | --- | --- | --- | --- |
| A | B | C |  | A | B | C |  | A | B | C |
| 15 | *Chryseobacterium* | 0.51 |  | 5 | *Pseudomonas* | 0.62 |  | 2 | *Erwinia* | 0.56 |
| 4 | *Pseudomonas viridiflava* | 0.41 |  | 17 | Xanthomonadaceae | 0.44 |  | 3 | *Bacillus flexus* | 0.41 |
| 17 | Xanthomonadaceae | 0.32 |  | 31 | *Achromobacter* | 0.24 |  | 5 | *Pseudomonas* | 0.41 |
| 221 | *Pseudomonas* | 0.27 |  | 26 | *Acinetobacter* | 0.24 |  | 6 | *Streptomyces* | 0.17 |
| 2 | *Erwinia* | 0.26 |  | 15 | *Chryseobacterium* | 0.20 |  | 10 | Kineosporiaceae | 0.15 |
| 20 | *Chryseobacterium* | 0.22 |  | 48 | *Sphingobacterium faecium* | 0.15 |  | 11 | Micromonosporaceae | 0.13 |
| 136 | *Janthinobacterium* | 0.20 |  | 75 | Comamonadaceae | 0.14 |  | 4 | *Pseudomonas viridiflava* | 0.13 |
| 7 | Aeromonadaceae | 0.18 |  | 4 | *Pseudomonas viridiflava* | 0.13 |  | 27 | *Streptomyces* | 0.11 |
| 49 | *Wautersiella* | 0.16 |  | 221 | *Pseudomonas* | 0.13 |  | 13 | *Caulobacter henricii* | 0.10 |
| 31 | *Achromobacter* | 0.16 |  | 2329 | Enterobacteriaceae | 0.12 |  | 23 | Actinomycetales | 0.10 |
